# The Neurologic Manifestations of Coronavirus Disease 2019 Pandemic: A Systemic Review

**DOI:** 10.3389/fneur.2020.00498

**Published:** 2020-05-19

**Authors:** Sheng-Ta Tsai, Ming-Kuei Lu, Shao San, Chon-Haw Tsai

**Affiliations:** ^1^Department of Neurology, China Medical University Hospital, Taichung, Taiwan; ^2^College of Medicine, China Medical University, Taichung, Taiwan; ^3^Graduate Institute of Acupuncture Science, College of Chinese Medicine, China Medical University, Taichung, Taiwan; ^4^Everflourish Neuroscience and Brain Disease Center, China Medical University Hospital, Taichung, Taiwan; ^5^Department of Anesthesiology, China Medical University Hospital, Taichung, Taiwan

**Keywords:** COVID-19, pandemic, neurologic, headache, taste, olfactory, ACE2, cytokine

## Abstract

**Objective:** Review and integrate the neurologic manifestations of the Coronavirus Disease 2019 (COVID-19) pandemic, to aid medical practitioners who are combating the newly derived infectious disease.

**Methods:** We reviewed the clinical research, consisting of mainly case series, on reported neurologic manifestations of COVID-19. We also reviewed basic studies to understand the mechanism of these neurologic symptoms and signs.

**Results:** We included 79 studies for qualitative synthesis and 63 studies for meta-analysis. The reported neurologic manifestations were olfactory/taste disorders (35.6%), myalgia (18.5%), headache (10.7%), acute cerebral vascular disease (8.1%), dizziness (7.9%), altered mental status (7.8%), seizure (1.5%), encephalitis, neuralgia, ataxia, Guillain-Barre syndrome, Miller Fisher syndrome, intracerebral hemorrhage, polyneuritis cranialis, and dystonic posture.

**Conclusions:** Neurologic manifestations in COVID-19 may alert physicians and medical practitioners to rule in high-risk patients. The increasing incidence of olfactory/taste disorders, myalgia, headache, and acute cerebral vascular disease renders a possibility that COVID-19 could attack the nervous system. The cytokine secretion and bloodstream circulation (viremia) are among the most possible routes into the nervous system.

## Introduction

COVID-19 first occurred in late 2019 in Wuhan, China (1). As of May 01, 2020, the COVID-19 pandemic had infected 3,291,008 worldwide and caused 232,478 deaths (data from the World Health Organization). The most common clinical symptoms are cough, sputum production, fatigue, shortness of breath, and mainly respiratory tract symptoms. However, an increasing number of cases have presented with neurologic manifestations, such as olfactory and taste disorders (2), and the phenomenon requires further attention.

COVID-19 is a new RNA virus strain from the family Coronaviridae (including the Middle East respiratory syndrome CoV [MERS-CoV] and severe acute respiratory syndrome CoV [SARS-CoV]). Phylogenetic analysis of the complete viral genome revealed that the virus was most closely related (89.1% nucleotide similarity) to a group of SARS-like coronaviruses (3). As such, it was previously termed SARS-CoV-2. In the review article published in 2018 (4), researchers found that the human coronavirus can enter the central nervous system through the olfactory bulb, causing demyelination and inflammation (cultured glial cells have been described to secrete cytokines including IL-6, IL-12p40, IL-15, TNF-a, CXCL9, and CXCL10 upon viral infection). The authors of a recent article (5) investigated the mechanism of COVID-19 nervous system involvement, and they stated that similar to SARS-CoV, the COVID-19 virus exploits the angiotensin-converting enzyme 2 (ACE2) receptor to gain entry inside the cells. The brain has been reported to express ACE2 receptors that have been detected over glial cells and neurons, which makes them a potential target of COVID-19. Recently, the research team in Harvard Medical School identified three main cells co-expressing ACE2 and TMPRSS2 (Type II transmembrane serine protease): lung type II pneumocytes, ileal absorptive enterocytes, and nasal goblet secretory cells (6). And the other research team used single-cell RNA-Seq datasets to suggest possible mechanisms through which CoV-2 infection could lead to anosmia or other forms of olfactory dysfunction (7).

## Methods

We searched the MEDLINE, CENTRAL, and EMBASE databases for eligible publications from December 2019 to April 30, 2020 written in English, using the following keywords: COVID-19, SARS-CoV-2, neuro, clinical, characteristics, manifestations. We also checked the reference lists of relevant studies to identify any missing publications. We reviewed the clinical researches, including case series and case reports, for neurologic manifestations of COVID-19 and organized them into tables. A confirmed case of COVID-19 (SARS-CoV-2) was defined and mostly diagnosed using the triple algorithm (epidemiological history, clinical symptoms, and laboratory or radiological findings) as a standard procedure proposed by the World Health Organization. We also reported data from the Taiwan Centers for Disease Control until May 01, 2020. Then we did the meta-analysis of all the case series to pool the data together and make it easier to understand. We used the software of Comprehensive Meta-Analysis Software (CMA), version 3, and chose the model of one group event rate, random effect, to draw the Forest Plot (Supplementary Figures 2–8).

## Results

We used Preferred Reporting Items for Systematic reviews and Meta-Analyzes (PRISMA) guidelines for searching and listed our flowchart (Supplementary Figure 1). Then we made a list of the neurologic manifestations in the current COVID-19 pandemic (Table 1). We included 9 case series and 4 case reports of olfactory or taste disorders. We pooled the case series together and found around 35.6% of patients got these symptoms (Supplementary Figure 2). We included 43 studies of myalgia, about 18.5% of patients had this symptom (Supplementary Table 1 and Supplementary Figure 3). And 45 studies of headache, the percentage was 10.7% (Supplementary Table 2 and Supplementary Figure 4); 2 studies of acute cerebral vascular disease, the percentage was 8.1% (Supplementary Figure 5); 7 studies of dizziness, the percentage was 7.9% (Supplementary Figure 6); 4 case series and 2 case reports of altered mental status, the percentage was 7.8% (Supplementary Figure 7); and 2 studies of seizure, the percentage was 1.5% (Supplementary Figure 8). And still other case reports of encephalitis, neuralgia, ataxia, Guillain-Barre syndrome, Miller Fisher syndrome, intracerebral hemorrhage, polyneuritis cranialis, and dystonic posture.

**Table 1:** List of the neurologic manifestations in the current COVID-19 pandemic.

**Neurologic manifestation**	**Patient numbers** **(% in total participants)**	**Total participants**	**Age:** **mean [SD] or median [IQR]**	**Published journal ^reference^**
With olfactory or/and taste disorders	20 (33.9%)	59	60 [50–74]	Clin Infect Dis (8)
	53 (12.4%)	429	32 [4–88]	Taiwan CDC (9)
	128 (75.7)	169	43 [34–54]	Int Forum Allergy Rhinol (10)
	25 (20%)	126	43.5 [3–87]	Trav Med Infect Dis (11)
	62 (19.4%)	320	No data	Laryngoscope (12)
	31 (39.2%)	79	61.6 [17.4]	Eur J Neurol (13)
	130 (64.4%)	202	56 [45–67]	JAMA (14)
	1	Case report	80	Eur J Case Rep Intern Med (15)
	1	Case report	50	Neurology (16)
Olfactory disorder only	3 (5.1%)	59	60 [50–74]	Clin Infect Dis (8)
	11 (5.1%)	214	52.7 [15.5]	JAMA Neurol (2)
	357 (85.6%)	417	36.9 [11.4]	EUR ARCH OTO-RHINO-L (17)
	1	Case report	85	Eur J Case Rep Intern Med (15)
Taste disorder only	5 (8.5%)	59	60 [50–74]	Clin Infect Dis (8)
	12 (5.6%)	214	52.7 [15.5]	JAMA Neurol (2)
	342 (82%)	417	36.9 [11.4]	EUR ARCH OTO-RHINO-L (17)
	1	Case report	39	Neurology (16)
Dizziness	1 (12.5%)	8	48.1 [13–76]	Clin Infect Dis (18)
	13 (9.4%)	138	56 [42–68]	JAMA (19)
	37 (8.1%)	452	58 [47–67]	Clin Infect Dis (20)
	21 (8%)	274	62 [44–70]	BMJ (21)
	5 (7%)	69	42 [35–62]	Clin Infect Dis (22)
	1 (4.17%)	24	32.5 [5–95]	Sci China Life Sci (23)
	2 (2%)	81	49.5 [11]	Lancet (24)
Altered mental status	9 (52.9%)	17	86.5 [68.6–97.]	J Infect (25)
	9 (9%)	99	55.5 [21–88]	Lancet (26)
	1 (5.9%)	17	75 [48–89]	J Med Virol (27)
	3 (0.7%)	452	58 [47–67]	Clin Infect Dis (20)
	1	Case report	No data	Radiology (28)
	1	Case report	74	J Med Virol (29)
Seizure	1 (4.8%)	21	70 [43–92]	JAMA (30)
	1 (0.5%)	214	52.7 [15.5]	JAMA Neurol (2)
Acute cerebrovascular disease	3 (23%)	13	63	N Engl J Med (31)
	6 (2.8)	214	52.7 [15.5]	JAMA Neurol (2)
	5	No data	40.4 [5.6]	N Engl J Med (32)
Neuralgia	5 (2.3%)	214	52.7 [15.5]	JAMA Neurol (2)
Ataxia	1 (0.5%)	214	52.7 [15.5]	JAMA Neurol (2)
Guillain-Barre syndrome	5 (0.4%)	1,000–1,200	No data	N Engl J Med (33)
	1	Case report	61	Lancet Neurol (34)
	1	Case report	65	J Clin Neurosci (35)
	1	Case report	71	Neurol Neuroimmunol Neuroinflamm (36)
Encephalitis	1	Case report	24	Int J Infect Dis (37)
	1	Case report	56	Travel Med Infect Dis (38)
	1	Case report	74	Cureus (39)
	1	Case report	No data	Brain Behav Immun (40)
	1	Case report	41	Brain Behav Immun (41)
Intracerebral hemorrhage	1	Case report	79	New Microbes New Infect (42)
Miller Fisher Syndrome	1	Case report	50	Neurology (16)
Polyneuritis cranialis	1	Case report	39	Neurology (16)
Sustained upward gaze, dystonic bilateral leg extension and altered responsiveness	1	Case report	6 week	Neurology (43)

**Figure 1:**
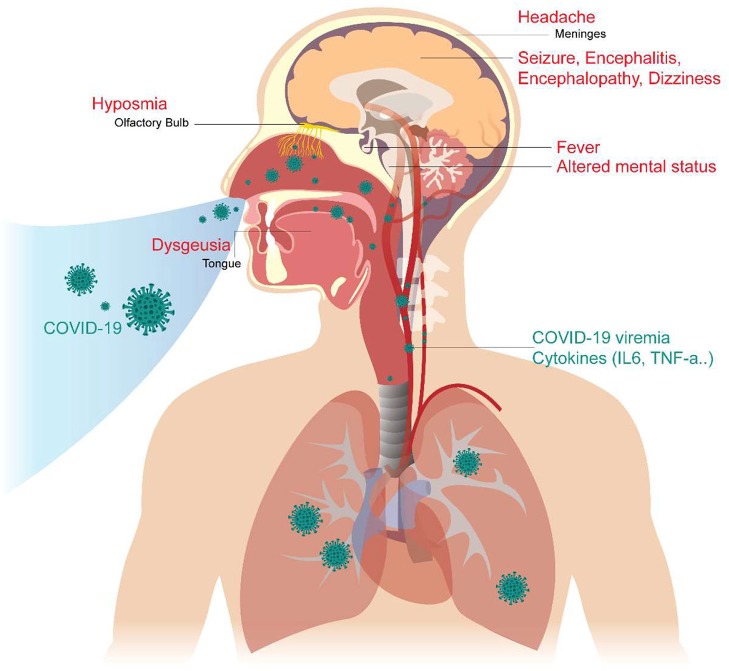
Neurological Manifestations of COVID-19 and the proposed mechanism. The COVID-19 virus may cause neurologic manifestations by cytokines secretion, general circulation (viremia), or direct invasion via the numerous ACE2 receptors in the olfactory epithelium. The olfactory disorder may cause by the olfactory epithelium damage. Fever was believed to be caused by the effect of cytokines or hypothalamus functional pertubation. The seizure may cause by cytokines storm, severely illed condition, or the brain parenchyma involvement, especially the mesial temporal lobe. Altered mental status may be a consequence of multiple organ failure, severe infection, or brainstem involvement. Headache is caused by meningeal irritation.

In addition, we reviewed some basic studies (4, 5, 44, 45) to determine the mechanism of these neurologic symptoms and signs. The cartoon figure summarized the possible mechanism (Figure 1).

## Discussion

The COVID-19 pandemic is currently progressing, and neurologists and medical practitioners worldwide will face additional challenges from the neurologic complications of the disease (46). An updated review focusing on the neurologic features may help clinicians early identify potential patients.

Interestingly, previous coronavirus infections, including MERS and SARS, did not have a large proportion of patients with olfactory and taste disorders (47). However, patients with COVID-19 frequently complain of abnormalities in smell and taste. In our analysis of data from Taiwan (9), we found that between January 21 and March 24, 2020, a total of 216 patients were confirmed to have COVID-19 infection, and 5 of them (2.3%) had olfactory or taste disorders. Between March 25 and May 01, 48 cases in 213 patients (22.5%) had olfactory or taste disorders. In the beginning, most COVID-19 patients had a contact history related to Wuhan. But after the government of China locked down many big cities, Taiwan's COVID-19 cases mostly originated from travelers from Europe, the Middle East, or the United States. Besides, according to 88 cases series (see Supplementary Tables 1, 2) in China (from December 2019 to April 25, 2020), only one study (2) conducted by neurologists in Wuhan reported olfactory or taste disorder.

On the other hand, an Italian researcher reported that 33.9% of COVID-19 patients in Italy experienced this problem (8). In the Middle East, researchers in Iran found a surge in the outbreak of olfactory dysfunction during the COVID-19 epidemic (based on an online checklist of 10,069 voluntary cases between March 12 and 17, 2020) (48). The different incidence of the olfactory and/or taste dysfunction by the timing and geographic distribution might reveal important information that the virus may carry the potential to alter its affinity to the central nervous system (49–51). However, the possibilities of a higher detection rate of olfactory dysfunction in patients diagnosed by certain sub-specialists, such as neurologists (2) or otolaryngologists, cannot be completely excluded. For example, the study conducted by otolaryngologists (17) found olfactory/taste disorders in more than 80% of the patients.

Fever is generally known as an elevation in body temperature caused by a cytokine-induced upward displacement of the set point of the hypothalamic thermoregulatory center. Small elevations in body temperature appear to enhance immune function and inhibit pathogen growth (52). In 2005, pathologists in Beijing performed autopsies of SARS patients and found signals of the SARS viral genome detected in numerous neurons in the hypothalamus (53). As a result, it is conceivable that fever may be caused mainly by the effect of cytokines or possible direct viral invasion to the hypothalamus.

Concerning seizure in viral infection, generally the paroxysmal spell may be a consequence of multiple complications of systemic disease, such as metabolic disturbances, hypoxia, etc. Considering the viral encephalitis, it frequently manifests with seizures in its acute phase (54). The most widely reported virus was HSV-1 (herpes simplex virus), which involves the highly epileptogenic mesial temporal lobe structures, including the hippocampus (54). In the two case reports (37, 39), both had mesial temporal lobe involvement (one by acute inflammation, one by previous ischemic stroke). Since the case number is limited, we can only speculate that seizures may be caused by the generalized poor condition, cytokine storm (55), or mesial temporal lobe involvement in severe COVID-19 patients.

Several countries are currently encountering a crisis of ventilator shortage. The respiratory failure of COVID-19 infected patients may be partly related to brainstem failure. The COVID-19 virus passes into the cell via the ACE2 receptor (5). ACE2 is expressed in the brain and is mainly found in the brainstem, specifically in the nuclei associated with cardio-respiratory control (56, 57). In the previous research on SARS-CoV-1 and MERS-COV, the brainstem was severely infected, which possibly contributes to the degradation and failure of respiratory centers (45). Besides, the ascending reticular activating system (ARAS), which is responsible for human consciousness, also originates from the brainstem (and then advances into the thalamus and cortex) (58). This may partly explain the altered mental status of COVID-19 patients. However, the maintenance of consciousness is complex. Considering many COVID-19 patients were severely ill with multi-organ failure, both the cytokine effect and systemic impact of organ dysfunction can also lead to the consciousness disturbance.

Both dizziness and headache are considered to be general non-specific symptoms. Etiologies attributed to infectious causes are important secondary causes of headache (59). It is known that cytokines induced by viral infection increase the permeability of vessels. This causes cerebral swelling and meningeal irritation. The meningeal irritation stimulates the trigeminal nerve terminals and triggers pain sensation (60).

Ischemic stroke also occurs in COVID-19 patients because the infection may cause D-dimer elevation, thrombocytopenia, and hypercoagulable state (61–66). Besides, the exaggerated systemic inflammation or a “cytokine storm” (55), cardioembolism from virus-related cardiac injury (67) could further increase the risk of stroke (68).

Most cases of Guillain-Barre syndrome appeared with a lag time from the primary infection of COVID-19 (33, 34); the pathogenesis is therefore likely to be postinfectious immune-mediated.

This review is obviously constrained by the current information and limited reports. And there was considerable heterogeneity in the data. In addition, the researches of the novel pandemic emerge fastly. We could only review the results up to April 30, 2020 in this regard. The cause of neurologic manifestation may be a cytokine storm, multiple organ failure, or direct viral infection. However, the detailed pathophysiology of causing COVID-19 nervous system involvement remains to be elucidated. We sincerely hope the review can help the first line clinicians identify the emerging neurologic manifestations when combating the viral pandemic.

## Author Contributions

S-TT and M-KL did the literature search and drafted this manuscript. C-HT initiated this review and integrated the clinical and basic research. SS did the meta-analysis and made all the Forest Plot figures.

## Conflict of Interest

The authors declare that the research was conducted in the absence of any commercial or financial relationships that could be construed as a potential conflict of interest.

## Supplementary Material

Supplementary Figure 1 Preferred Reporting Items for Systematic reviews and Meta-Analyzes (PRISMA), our searching strategy.

Supplementary Figure 1 Forest plot for olfactory/taste disorder.

Supplementary Figure 3 Forest plot for myalgia.

Supplementary Figure 4 Forest plot for headache.

Supplementary Figure 5 Forest plot for acute cerebral vascular disease.

Supplementary Figure 6 Forest plot for dizziness.

Supplementary Figure 7 Forest plot for altered mental status.

Supplementary Figure 8 Forest plot for seizure.

Supplementary Table 1 Patients presented with myalgia.

Supplementary Table 2 Patients presented with headache (ordered by cases recruitment date).
