# Supplementary material for: The Neurologic Manifestations of Coronavirus Disease 2019 Pandemic: A Systemic Review
Source: Front Neurol. 2020 May 19;11:498. doi: 10.3389/fneur.2020.00498 (PMC7248254; doi:10.3389/fneur.2020.00498)

Supplementary Figure 1. Preferred Reporting Items for Systematic reviews and Meta-Analyzes (PRISMA), our searching strategy


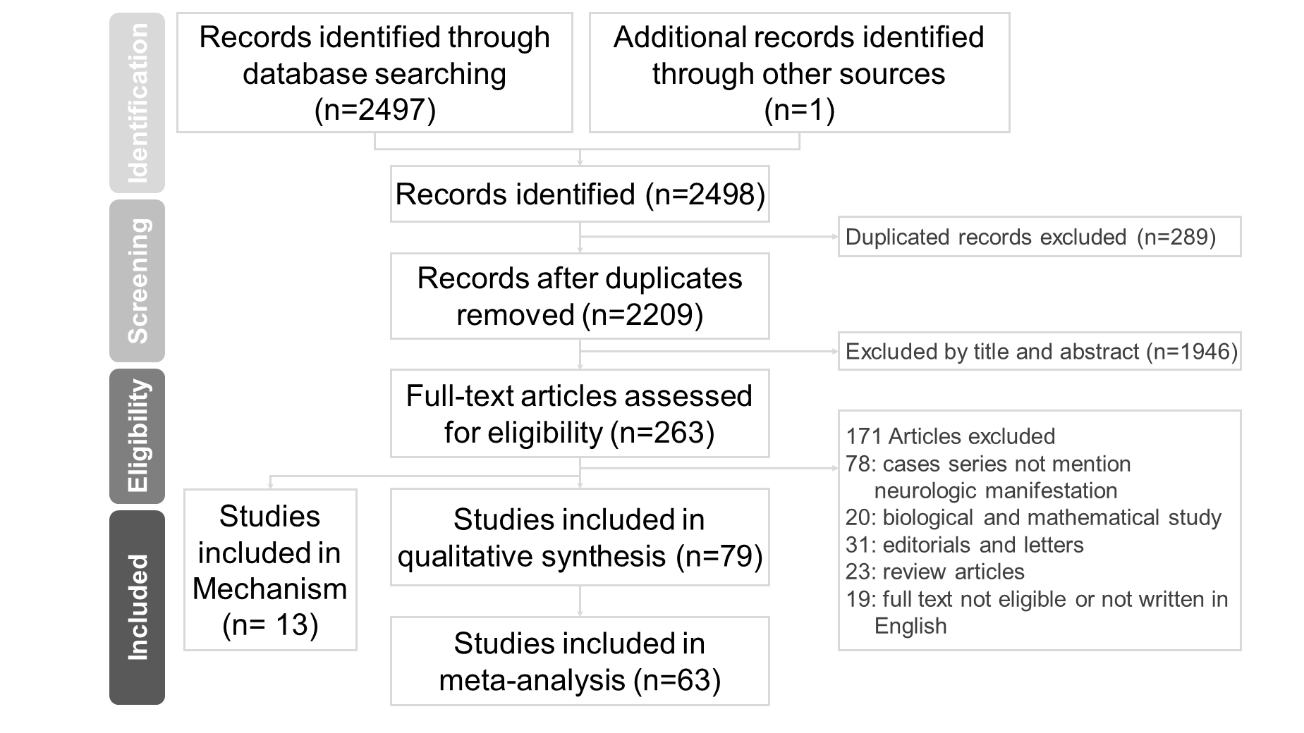


Supplementay Figure 2. Forest plot for olfactory/taste disorder


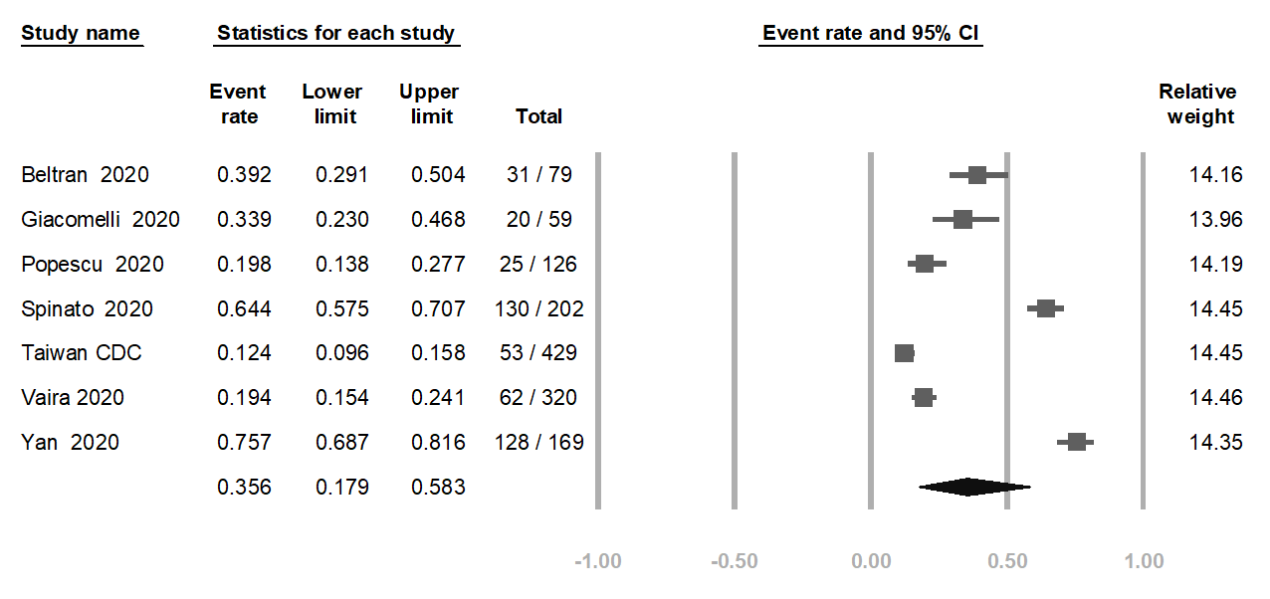


Supplementary Figure 3. Forest plot for myalgia


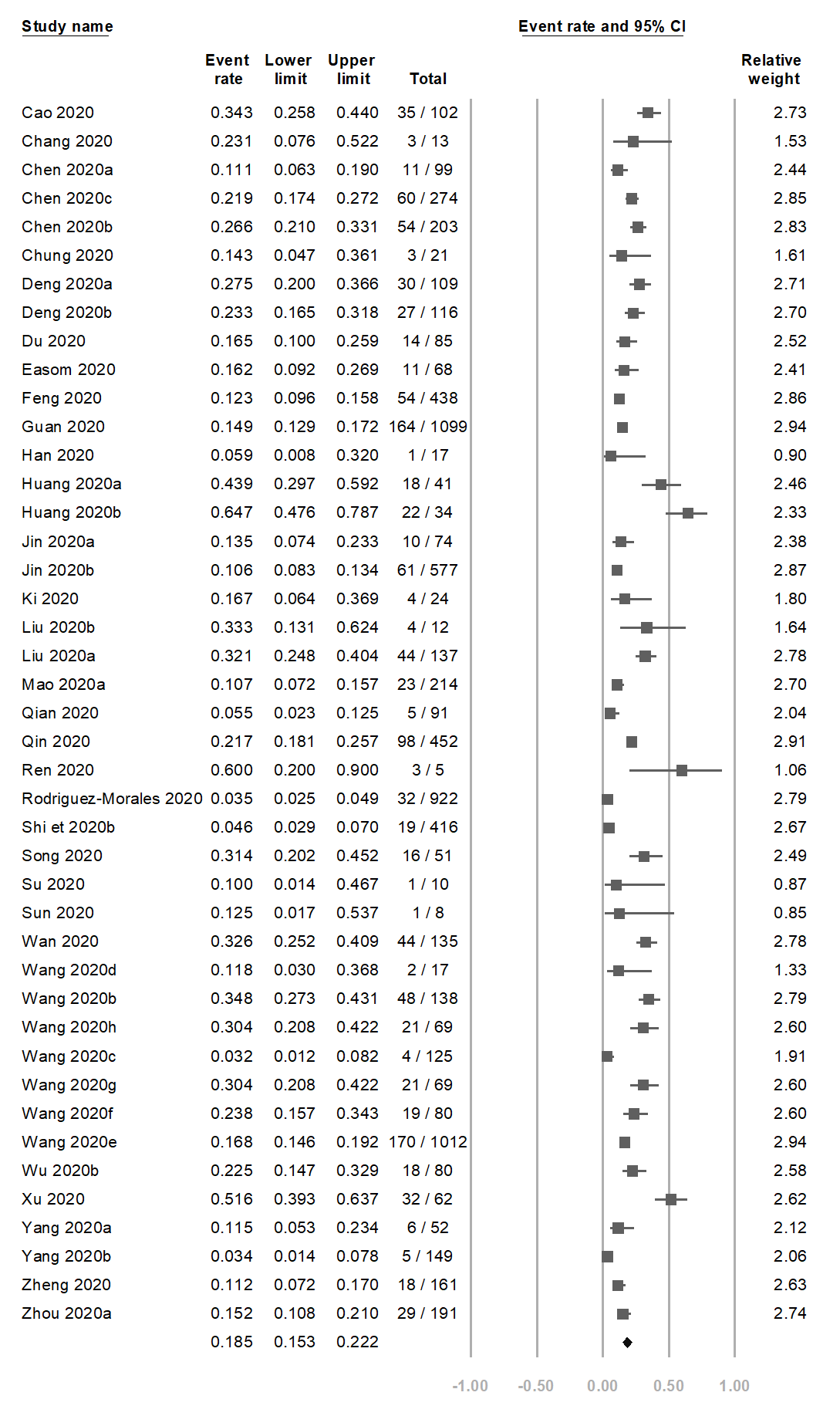


Supplementary Figure 4. Forest plot for headache


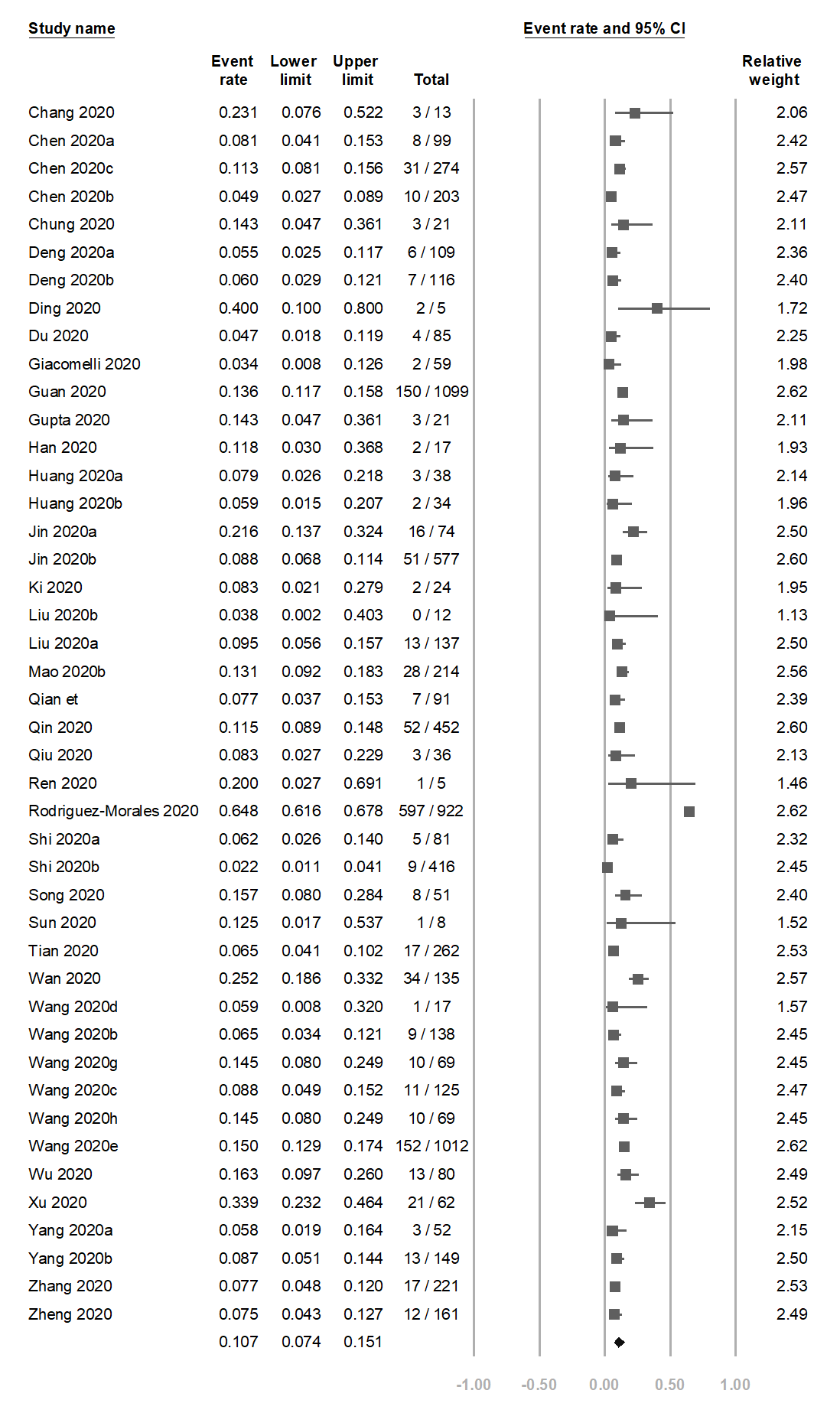


Supplementary Figure 5. Forest plot for acute cerebral vascular disease


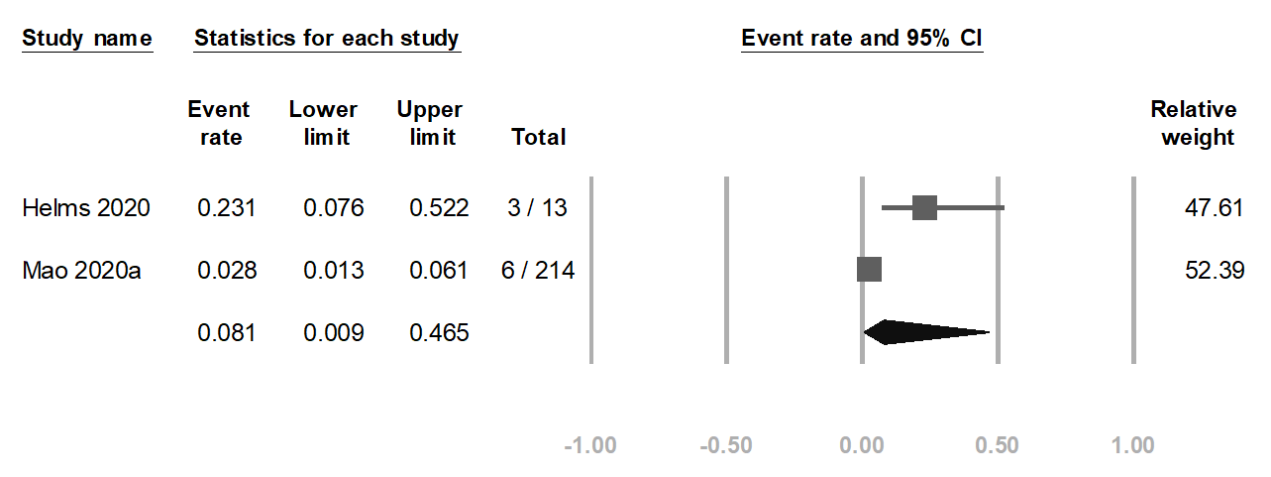


Supplementary Figure 6. Forest plot for dizziness


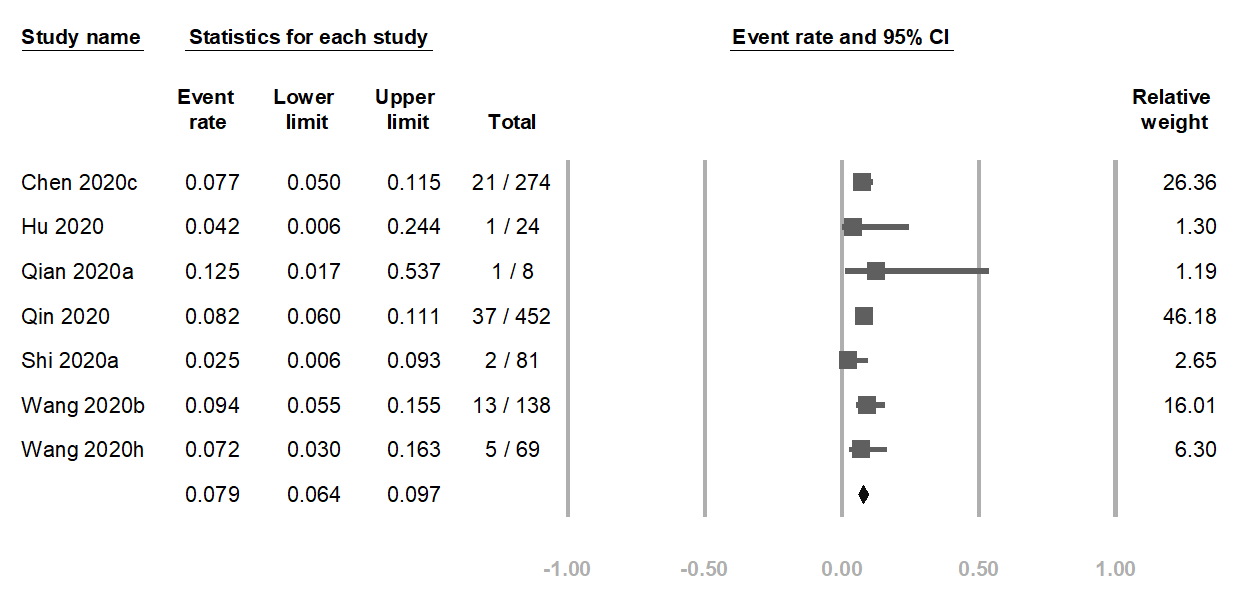


Supplementary Figure 7. Forest plot for altered mental status


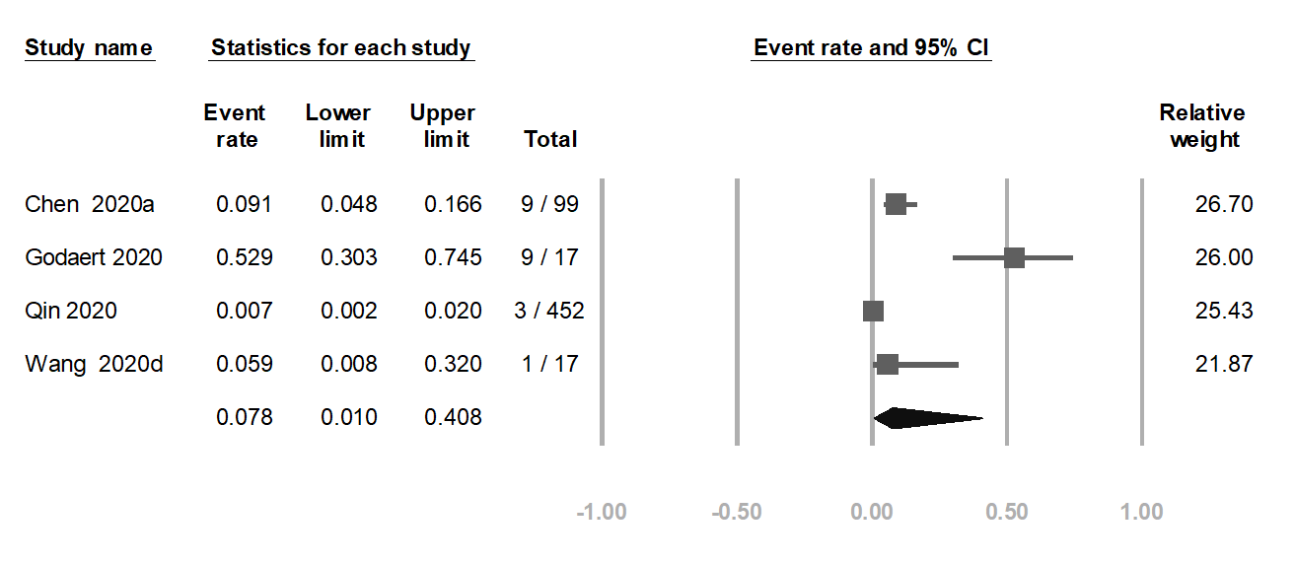


Supplementary Figure 8. Forest plot for seizure


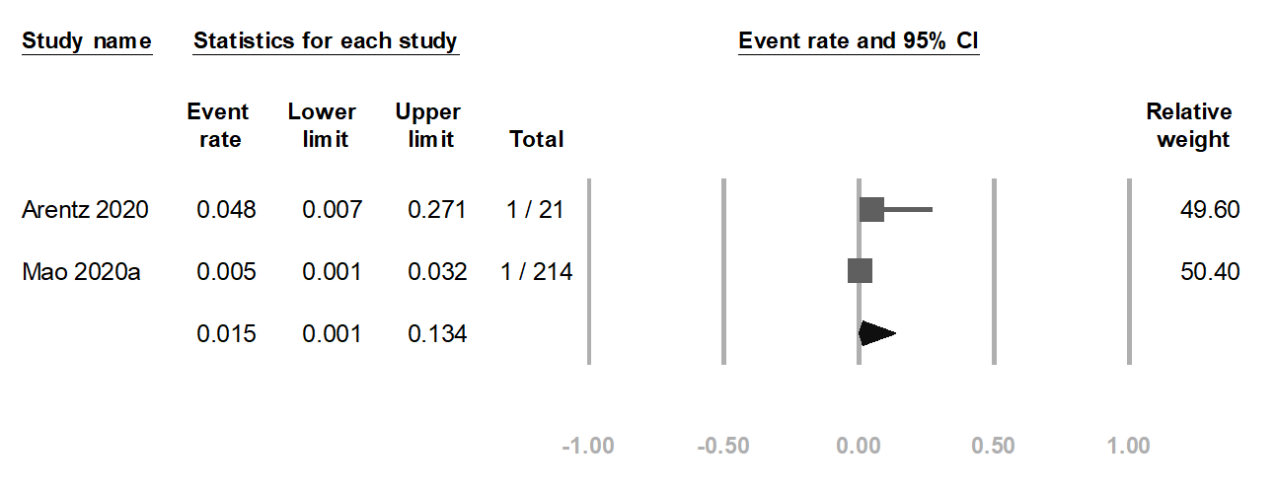

Supplement: Supplementary Figure 1 — Preferred Reporting Items for Systematic reviews and Meta-Analyzes (PRISMA), our searching strategy. [file Data_Sheet_2.docx]
