# Supplementary material for: The Neurologic Manifestations of Coronavirus Disease 2019 Pandemic: A Systemic Review
Source: Front Neurol. 2020 May 19;11:498. doi: 10.3389/fneur.2020.00498 (PMC7248254; doi:10.3389/fneur.2020.00498)
Supplement: Supplementary Table 1 — Patients presented with myalgia. [file Data_Sheet_1.docx]

**Supplementary Table 1. Patients presented with myalgia**

| **No** | **Patient numbers (% in total participants)** | **Total participants** | **Age:**  **mean [SD] or median [IQR]** | **Published journal (reference)** |
| --- | --- | --- | --- | --- |
| 1 | 164 (14.9%) | 1099 | 47 [35-58] | N Engl J Med (70) |
| 2 | 2 (11.8%) | 17 | 75 [48-89] | J Med Virol (56) |
| 3 | 18 (44%) | 41 | 49 [41-58] | Lancet (71) |
| 4 | 3 (60%) | 5 | 53.6 [41-65] | Chin Med J (72) |
| 5 | 1 (5.9%) | 17 | 40 [9.6] | Clin Infect Dis (73) |
| 6 | 4 (33.3%) | 12 | 62.5 [10-72] | Sci China Life Sci (74) |
| 7 | 6 (11.5%) | 52 | 59.7 [13.3] | Lancet (75) |
| 8 | 44 (32.1%) | 137 | 55 [20-83] | Chin Med J (76) |
| 9 | 11 (11%) | 99 | 55.5 [21-88] | Lancet (55) |
| 10 | 48 (34.8%) | 138 | 56 [42-68] | JAMA (48) |
| 11 | 98 (21.7%) | 452 | 58 [47-67] | Clin Infect Dis (49) |
| 12 | 32 (52%) | 62 | 41 [32-52] | BMJ (77) |
| 13 | 60 (22%) | 274 | 62 [44-70] | BMJ (50) |
| 14 | 3 (23.1%) | 13 | 34 [34-48] | JAMA (78) |
| 15 | 21 (30%) | 69 | 42 [35-62] | Clin Infect Dis (51) |
| 16 | 23 (10.7%) | 214 | 52.7 [15.5] | JAMA Neurology (2) |
| 17 | 10 (13.51%) | 74 | 46.1 [14.2] | Gut (79) |
| 18 | 61 (10.57%) | 577 | 45.1 [14.45] | Gut (79) |
| 19 | 3 (14%) | 21 | 51 [29-77] | Radiology (80) |
| 20 | 16 (31%) | 51 | 49 [16] | Radiology (81) |
| 21 | 19 (4.6%) | 416 | 64 [21-95] | JAMA Cardiology (82) |
| 22 | 1 (12.5%) | 8 | 2m to 15y | World J Pediatr (83) |
| 23 | 4 (16.7%) | 24 | 42 [21-62] | Epidemiol Health (84) |
| 24 | 54 (26.6%) | 203 | 54 [20-91] | J Gerontol (85) |
| 25 | 54 (12.6%) | 438 | 53 [40-64] | Am J Resp Crit Care (86) |
| 26 | 1 (10%) | 10 | No data | Travel Med Infect (87) |
| 27 | 4 (3.2%) | 125 | 38.8 [13.8] | Int J Infect Dis (88) |
| 28 | 18 (22.5%) | 80 | 46.1 [15.4] | Clin Infect Dis (89) |
| 29 | 22 (64.7%) | 34 | 56.2 [17.1] | Travel Med Infect (90) |
| 30 | 21 (30%) | 69 | 42 [35-62] | Clin Infect Dis (91) |
| 31 | 19 (23.75%) | 80 | 39 [32-48.5] | J Hosp Infect (92) |
| 32 | 14 (16.5%) | 85 | 65.8 [14.2] | Am J Resp Crit Care (93) |
| 33 | 35 (34.3%) | 102 | 54 [37-67] | Clin Infect Dis (94) |
| 34 | 170 (16.8%) | 1012 | 50 [39-58] | Clin Microbiol Infect (95) |
| 35 | 30 (27.5%) | 109 | 69 [62-74] | Chin Med J (96) |
| 36 | 27 (23.3%) | 116 | 40 [33-57] | Chin Med J (96) |
| 37 | 18 (11.2%) | 161 | 45 [33.5-57] | Eur Rev Med Pharmaco (97) |
| 38 | 11 (16%) | 68 | 42.4 | Influenza Other Resp (98) |
| 39 | 44 (32.5%) | 135 | 47 [36-55] | J Med Virol (99) |
| 40 | 5 (5.49%) | 91 | 50 [36.5-57] | QJM (100) |
| 41 | 32 (3.5%) | 922 | No data | Curr Trop Med Rep (17) |
| 42 | 5 (3.36%) | 149 | 45.1 [13.35] | J Infect (101) |
| 43 | 29 (15%) | 191 | 56 [46-67] | Lancet (102) |

|  |  |  |  |  |
| --- | --- | --- | --- | --- |

**Supplementary Table 2. Patients presented with headache (ordered by cases recruitment date)**

| **No^a^** | **Patient numbers (% in total participants)** | **Total participants** | **Age:**  **mean [SD] or median [IQR]** | **Published journal (reference)** |
| --- | --- | --- | --- | --- |
| 1 | 150 (3.6%) | 1099 | 47 [35-58] | N Engl J Med (70) |
| 2 | 1 (5.9%) | 17 | 75 [48-89] | J Med Virol (56) |
| 3 | 3 (8%) | 38 | 49 [41-58] | Lancet (71) |
| 4 | 1 (20%) | 5 | 53.6 [41-65] | Chin Med J (Engl) (72) |
| 5 | 5 (6%) | 81 | 49.5 [11] | Lancet (53) |
| 6 | 2 (11.8%) | 17 | 40 [9.6] | Clin Infect Dis (73) |
| 7 | 0 (0%) | 12 | 62.5 [10-72] | Sci China Life Sci (74) |
| 8 | 3 (6%) | 52 | 59.7 [13.3] | Lancet (75) |
| 9 | 2 (40%) | 5 | 50.2 [9.8] | J Med Virol (103) |
| 10 | 13 (9.5%) | 137 | 55 [20-83] | Chin Med J (Engl) (76) |
| 11 | 8 (8%) | 99 | 55.5 [21-88] | Lancet (55) |
| 12 | 9 (6.5%) | 138 | 56 [42-68] | JAMA (48) |
| 13 | 52 (11.4%) | 452 | 58 [47-67] | Clin Infect Dis (49) |
| 14 | 21 (34%) | 62 | 41 [32-52] | BMJ (77) |
| 15 | 31 (11%) | 274 | 62 [44-70] | BMJ (50) |
| 16 | 3 (23.1%) | 13 | 34 [34-48] | JAMA (78) |
| 17 | 10 (14%) | 69 | 42 [35-62] | Clin Infect Dis (51) |
| 18 | 28 (13.1%) | 214 | 52.7 [15.5] | JAMA Neurol (2) |
| 19 | 16 (21.62%) | 74 ^b^ | 46.14 [14.19] | Gut (79) ^a^ |
|  | 51 (8.84%) | 577 ^c^ | 45.09 [14.45] | Gut (79) ^b^ |
| 20 | 3 (8.3%) | 36 | 8.3 [3.5] | Lancet Infect Dis (104) |
| 21 | 3 (14%) | 21 | 51 [29-77] | Radiology (80) |
| 22 | 8 (16%) | 51 | 49 [16] | Radiology (81) |
| 23 | 9 (2.2%) | 416 | 64 [21-95] | JAMA Cardiol (82) |
| 24 | 1 (12.5%) | 8 | 2m to 15y ^c^ | World J Pediatr (83) |
| 25 | 2 (8.3%) | 24 | 42 [21-62] | Epidemiol Health (84) |
| 26 | 2 (3.4%) | 59 | 60 [50-74] | Clin Infect Dis (13) |
| 27 | 10 (4.9%) | 203 | 54 [20-91] | J Gerontol (85) |
| 28 | 17 (6.5%) | 262 | 47.5 [1-94] | J Infect (105) |
| 29 | 3 (13.6%) | 21 | 40.3 [16-73] | Travel Med Infect (106) |
| 30 | 11 (8.8%) | 125 | 38.8 [13.8] | Int J Infect Dis (88) |
| 31 | 13 (16.25%) | 80 | 46.1 [15.4] | Clin Infect Dis (89) |
| 32 | 2 (5.9%) | 34 | 56.2 [17.1] | Travel Med Infect (90) |
| 33 | 10 (14%) | 69 | 42 [35-62] | Clin Infect Dis (91) |
| 34 | 8 (10%) | 80 | 39 [32-48.5] | J Hosp Infect (92) |
| 35 | 4 (4.7%) | 85 | 65.8 [14.2] | Am J Resp Crit Care (93) |
| 36 | 17 (7.7%) | 221 | 55 [39-66.5] | J Clin Virol (107) |
| 39 | 152 (15%) | 1012 | 50 [39-58] | Clin Microbiol Infect (95) |
| 40 | 6 (5.5%) | 109 | 69 [62-74] | Chin Med J (96) |
| 41 | 7 (6%) | 116 | 40 [33-57] | Chin Med J (96) |
| 42 | 12 (7.5%) | 161 | 45 [33.5-57] | Eur Rev Med Pharmaco (97) |
| 43 | 34 (25.2%) | 135 | 47 [36-55] | J Med Virol (99) |
| 44 | 7 (7.69%) | 91 | 50 [36.5-57] | QJM (100) |
| 45 | 597 (64.8%) | 922 | no data | Curr Trop Med Rep (17) |
| 46 | 13 (8.72%) | 149 | 45.1 [13.35] | J Infect (101) |
| 47 | 1 | Case report | 74 | Cureus (22) |
|  |  |  |  |  |

1. Patients with gastro-intestinal symptoms
2. Patients without gastro-intestinal symptoms
3. 2m= 2 months old; 15y= 15 years old

**References**

70. Guan W-j, Ni Z-y, Hu Y, Liang W-h, Ou C-q, He J-x, et al. Clinical Characteristics of Coronavirus Disease 2019 in China. N Engl J Med 2020; 382:1708-1720

71. Huang C, Wang Y, Li X, Ren L, Zhao J, Hu Y, et al. Clinical features of patients infected with 2019 novel coronavirus in Wuhan, China. The Lancet. 2020;395(10223):497-506.

72. Ren LL, Wang YM, Wu ZQ, Xiang ZC, Guo L, Xu T, et al. Identification of a novel coronavirus causing severe pneumonia in human: a descriptive study. Chin Med J (Engl). 2020 May 5;133(9):1015-1024.

73. Han X, Cao Y, Jiang N, Chen Y, Alwalid O, Zhang X, et al. Novel Coronavirus Pneumonia (COVID-19) Progression Course in 17 Discharged Patients: Comparison of Clinical and Thin-Section CT Features During Recovery. Clinical Infectious Diseases, ciaa271. doi: 10.1093/cid/ciaa271

74. Liu Y, Yang Y, Zhang C, Huang F, Wang F, Yuan J, et al. Clinical and biochemical indexes from 2019-nCoV infected patients linked to viral loads and lung injury. Sci China Life Sci. 2020;63(3):364-74.

75. Yang X, Yu Y, Xu J, Shu H, Xia Ja, Liu H, et al. Clinical course and outcomes of critically ill patients with SARS-CoV-2 pneumonia in Wuhan, China: a single-centered, retrospective, observational study. The Lancet Respiratory Medicine. 2020;8(5): 475-481

76. Liu K, Fang YY, Deng Y, Liu W, Wang MF, Ma JP, et al. Clinical characteristics of novel coronavirus cases in tertiary hospitals in Hubei Province. Chin Med J (Engl). 2020 May 5;133(9):1025-1031

77. Xu XW, Wu XX, Jiang XG, Xu KJ, Ying LJ, Ma CL, et al. Clinical findings in a group of patients infected with the 2019 novel coronavirus (SARS-Cov-2) outside of Wuhan, China: retrospective case series. BMJ. 2020 Feb 19;368:m606. doi: 10.1136/bmj.m606

78. Chang D, Lin M, Wei L, Xie L, Zhu G, Dela Cruz CS, et al. Epidemiologic and Clinical Characteristics of Novel Coronavirus Infections Involving 13 Patients Outside Wuhan, China. JAMA. 2020;323(11):1092-3.

79. Jin X, Lian J-S, Hu J-H, Gao J, Zheng L, Zhang Y-M, et al. Epidemiological, clinical and virological characteristics of 74 cases of coronavirus-infected disease 2019 (COVID-19) with gastrointestinal symptoms. Gut. 2020 Jun;69(6):1002-1009. doi: 10.1136/gutjnl-2020-320926

80. Chung M, Bernheim A, Mei X, Zhang N, Huang M, Zeng X, et al. CT Imaging Features of 2019 Novel Coronavirus (2019-nCoV). Radiology. 2020;295(1):202-7.

81. Song F, Shi N, Shan F, Zhang Z, Shen J, Lu H, et al. Emerging 2019 Novel Coronavirus (2019-nCoV) Pneumonia. Radiology. 2020;295(1):210-7.

82. Shi S, Qin M, Shen B, Cai Y, Liu T, Yang F, et al. Association of Cardiac Injury With Mortality in Hospitalized Patients With COVID-19 in Wuhan, China. JAMA Cardiol. Published online March 25, 2020. doi:10.1001/jamacardio.2020.0950

83. Sun D, Li H, Lu XX, Xiao H, Ren J, Zhang FR, et al. Clinical features of severe pediatric patients with coronavirus disease 2019 in Wuhan: a single center's observational study. World J Pediatr. 2020 Mar 19. doi: 10.1007/s12519-020-00354-4

84. Ki M. Epidemiologic characteristics of early cases with 2019 novel coronavirus (2019-nCoV) disease in Korea. Epidemiol Health. 2020;42:e2020007. doi: 10.4178/epih.e2020007

85. Chen T, Dai Z, Mo P, Li X, Ma Z, Song S, et al. Clinical characteristics and outcomes of older patients with coronavirus disease 2019 (COVID-19) in Wuhan, China (2019): a single-centered, retrospective study. The Journals of Gerontology: Series A, glaa089. doi: 10.1093/gerona/glaa089

86. Feng Y, Ling Y, Bai T, Xie Y, Huang J, Li J, et al. COVID-19 with Different Severity: A Multi-center Study of Clinical Features. Am J Respir Crit Care Med. 2020 Apr 10. doi: 10.1164/rccm.202002-0445OC

87. Su YJ, Lai YC. Comparison of clinical characteristics of coronavirus disease (COVID-19) and severe acute respiratory syndrome (SARS) as experienced in Taiwan. Travel Med Infect Dis. 2020 Mar 14:101625. doi: 10.1016/j.tmaid.2020.101625

88. Wang R, Pan M, Zhang X, Fan X, Han M, Zhao F, et al. Epidemiological and clinical features of 125 Hospitalized Patients with COVID-19 in Fuyang, Anhui, China. IJID. Published: April 11, 2020. doi: 10.1016/j.ijid.2020.03.070

89. Wu J, Liu J, Zhao X, Liu C, Wang W, Wang D, et al. Clinical Characteristics of Imported Cases of COVID-19 in Jiangsu Province: A Multicenter Descriptive Study. Clinical Infectious Diseases, ciaa199. doi: 10.1093/cid/ciaa199

90. Huang Y, Tu M, Wang S, Chen S, Zhou W, Chen D, et al. Clinical characteristics of laboratory confirmed positive cases of SARS-CoV-2 infection in Wuhan, China: A retrospective single center analysis. Travel Med Infect Dis. 2020 Feb 27:101606. doi: 10.1016/j.tmaid.2020.101606

91. Wang Z, Yang B, Li Q, Wen L, Zhang R. Clinical Features of 69 Cases with Coronavirus Disease 2019 in Wuhan, China. Clin Infect Dis. 2020 Mar 16. pii: ciaa272. doi: 10.1093/cid/ciaa272

92. Wang X, Liu W, Zhao J, Lu Y, Wang X, Yu C, et al. Clinical characteristics of 80 hospitalized frontline medical workers infected with COVID-19 in Wuhan, China. J Hosp Infect. 2020 Apr 14. pii: S0195-6701(20)30194-8. doi: 10.1016/j.jhin.2020.04.019

93. Du Y, Tu L, Zhu P, Mu M, Wang R, Yang P, et al. Clinical Features of 85 Fatal Cases of COVID-19 from Wuhan: A Retrospective Observational Study. Am J Respir Crit Care Med. 2020. doi: 10.1164/rccm.202003-0543OC

94. Cao J, Tu WJ, Cheng W, Yu L, Liu YK, Hu X, et al. Clinical Features and Short-term Outcomes of 102 Patients with Corona Virus Disease 2019 in Wuhan, China. Clinical Infectious Diseases, ciaa243. doi: 10.1093/cid/ciaa243

95. Wang X, Fang J, Zhu Y, Chen L, Ding F, Zhou R, et al. Clinical characteristics of non-critically ill patients with novel coronavirus infection (COVID-19) in a Fangcang Hospital. Clin Microbiol Infect. 2020 Apr 3. pii: S1198-743X(20)30177-4. doi: 10.1016/j.cmi.2020.03.032

96. Deng Y, Liu W, Liu K, Fang YY, Shang J, Zhou L, et al. Clinical characteristics of fatal and recovered cases of coronavirus disease 2019 (COVID-19) in Wuhan, China: a retrospective study. Chin Med J (Engl). 2020 Mar 20. doi: 10.1097/CM9.0000000000000824

97. Zheng F, Tang W, Li H, Huang YX, Xie YL, Zhou ZG. Clinical characteristics of 161 cases of corona virus disease 2019 (COVID-19) in Changsha. Eur Rev Med Pharmacol Sci. 2020;24(6):3404-10.

98. Easom N, Moss P, Barlow G, Samson A, Taynton T, Adams K, et al. Sixty-eight consecutive patients assessed for COVID-19 infection: Experience from a UK Regional infectious diseases Unit. Influenza Other Respir Viruses. 2020 Mar 29. doi: 10.1111/irv.12739

99. Wan S, Xiang Y, Fang W, Zheng Y, Li B, Hu Y, et al. Clinical features and treatment of COVID-19 patients in northeast Chongqing. J Med Virol. First published: 21 March 2020. doi: 10.1002/jmv.25783

100. Qian GQ, Yang NB, Ding F, Ma AHY, Wang ZY, Shen YF, et al. Epidemiologic and Clinical Characteristics of 91 Hospitalized Patients with COVID-19 in Zhejiang, China: A retrospective, multi-centre case series. QJM, hcaa089. doi: 10.1093/qjmed/hcaa089

101. Yang W, Cao Q, Qin L, Wang X, Cheng Z, Pan A, et al. Clinical characteristics and imaging manifestations of the 2019 novel coronavirus disease (COVID-19):A multi-center study in Wenzhou city, Zhejiang, China. J Infect. 2020;80(4):388-93.

102. Zhou F, Yu T, Du R, Fan G, Liu Y, Liu Z, et al. Clinical course and risk factors for mortality of adult inpatients with COVID-19 in Wuhan, China: a retrospective cohort study. The Lancet. 2020;395(10229):1054-62.

103. Ding Q, Lu P, Fan Y, Xia Y, Liu M. The clinical characteristics of pneumonia patients coinfected with 2019 novel coronavirus and influenza virus in Wuhan, China. J Med Virol. 2020 Mar 20. doi: 10.1002/jmv.25781

104. Qiu H, Wu J, Hong L, Luo Y, Song Q, Chen D. Clinical and epidemiological features of 36 children with coronavirus disease 2019 (COVID-19) in Zhejiang, China: an observational cohort study. The Lancet Infectious Diseases. Published:March 25, 2020. doi: 10.1016/S1473-3099(20)30198-5

105. Tian S, Hu N, Lou J, Chen K, Kang X, Xiang Z, et al. Characteristics of COVID-19 infection in Beijing. J Infect. 2020;80(4):401-6.

106. Gupta N, Agrawal S, Ish P, Mishra S, Gaind R, Usha G, et al. Clinical and epidemiologic profile of the initial COVID-19 patients at a tertiary care centre in India. Monaldi Arch Chest Dis. 2020 Apr 10;90(1). doi: 10.4081/monaldi.2020.1294

107. Zhang G, Hu C, Luo L, Fang F, Chen Y, Li J, et al. Clinical features and short-term outcomes of 221 patients with COVID-19 in Wuhan, China. J Clin Virol. 2020 Apr 9;127:104364. doi: 10.1016/j.jcv.2020.104364
